# Ig–microbiota binding patterns in mothers and infants: a scoping review

**DOI:** 10.1017/gmb.2026.10028

**Published:** 2026-06-01

**Authors:** Alicia Tan Yi Jia, Christopher S. Peacock, Danielle E. Dye, Claus T. Christophersen

**Affiliations:** 1 The Western Australian Human Microbiome Collaboration Centre, School of Molecular and Life Sciences, Curtin University, Bentley, WA, Australia; 2 Curtin Medical School, Curtin Medical Research Institute, Faculty of Health Sciences, Curtin University, Bentley, WA, Australia; 3 School of Biomedical Sciences, The University of Western Australia, Nedlands, WA, Australia; 4 Nutrition Health Innovation & Research Institute, School of Medical and Health Sciences, Edith Cowan University, Joondalup, WA, Australia

**Keywords:** Ig-Seq, cell sorting, sequencing, maternal, infant, microbiome, antibody–microbiota

## Abstract

Studies characterising the immunoglobulin (Ig)-bound microbiota apply varying methodologies, making comparisons difficult. This scoping review synthesised evidence on Ig–microbiota binding patterns in maternal and infant contexts, identified recurrent Ig-bound and -unbound bacteria across studies, and highlighted knowledge gaps for further study. Nine articles investigating Ig–microbiota binding patterns in stool or breastmilk samples in mothers or infants were included. Ig–microbiota associations were influenced by sample type, Ig-subclass, genetics, and diet. The most important antibody was IgA, with partial functional redundancy with IgM, while IgG appeared more selective for pathobionts. Ig-bound taxa in early life included important commensals and pathobionts, with high levels of individuality. Ig–microbiota associations shifted with microbiome maturation, environmental and host factors, resembling adults at around 2 years of age. Transfer of Ig-bound *Bifidobacterium* through breastmilk may contribute to vertical transmission from mother to infant. Ig–microbiota associations also differed between health and disease states, beyond the overall microbiota. Results were limited by study numbers and a lack of methodological consistency. We propose the standardised term “Ig-Seq” in referring to the technique to study Ig–microbiota binding patterns, and suggest standardisation of laboratory protocols, bioinformatic pipelines, and statistical analyses to improve consistency in Ig-Seq.

## Social media summary

Introducing Ig-Seq: Ig–microbiota associations in mothers and infants change with Ig subclass, age, diet and health.

## Introduction

The infant gut microbiome develops in tandem with the immune system in an interdependent manner, with development of both systems being affected by maternal and environmental factors (Gomez de Agüero et al., 2016; Koch et al., 2016; Timmerman et al., 2017; Al Nabhani and Eberl, 2020). Three critical periods in the infant gut microbiome development have previously been described – the prenatal, early postnatal (post-birth up to 6 months of age), and weaning periods (~6 months of age) (Hornef and Torow, 2020). These periods also correspond to critical windows in immune system development, particularly the neonatal and weaning periods (Al Nabhani and Eberl, 2020). Reduced microbial exposure or inappropriate stimulation during each of these periods has been associated with long-lasting consequences later in life, and greater risk of immune-mediated diseases such as allergic disease, cancer, and inflammatory bowel disease (Zimmermann et al., 2019; Al Nabhani and Eberl, 2020; Torres et al., 2020).

In the fetal period, the immune system starts to develop in a controlled manner, while protected by maternal transfer of IgG across the placenta (Ander et al., 2019). The most recent evidence suggests that the existence of a fetal microbiome is unlikely (Kennedy et al., 2023). However, there is a possibility of maternal metabolites produced by the maternal microbiome being transferred to the foetus via maternal antibodies, allowing for immune education during pregnancy in preparation for postnatal microbial exposure (Gomez de Agüero et al., 2016; Torow et al., 2023). This also prevents inappropriate early immune stimulation while in utero (Torow et al., 2023). This concept of protection in the form of maternal antibodies while the immune system is developing is also applied to the early postnatal period (Koch et al., 2016; Torow et al., 2023). The infant is exposed to microbes from the mother’s vaginal and perineal area during delivery, and this forms the start of the developing infant microbiome (Dominguez-Bello et al., 2019). The infant receives breastmilk, including microbes, maternal metabolites, and maternal antibodies (secretory IgA, IgG, and IgM) through breastfeeding (Fernández et al., 2013; Smilowitz et al., 2014; Timmerman et al., 2017; Cheng et al., 2021). This contributes to the infant microbiome and protection to maternal microbes they are exposed to while the immune system continues to develop, with breastmilk as the main food source in the early postnatal stage (Hornef and Torow, 2020; Torow et al., 2023). The weaning period constitutes cessation of breastfeeding and the introduction of solid foods (Al Nabhani and Eberl, 2020; Backhed et al., 2015; Hornef and Torow, 2020). This increased exposure to carbohydrate dietary antigens shifts the infant microbiome towards an adult-like composition with greater microbial diversity (Backhed et al., 2015). The infant immune system is also activated upon encountering an increased range of microbes and microbial metabolites, and starts to mature to simulate an adult (Al Nabhani and Eberl, 2020). This is a layered process, with maternal antibodies preventing early stimulation of the infant’s immune system while the infant microbiome is still developing (Al Nabhani and Eberl, 2020; Hornef and Torow, 2020). This prevents immune imprinting against the transient microbiota, which contains inflammatory taxa such as *Enterobacteriaceae* and hence induction of inflammatory responses against microbiota in subsequent exposures (c). Following weaning, homeostasis is regulated by the infant, with the production of regulatory T cells (Tregs) against food and microbiota antigens in the process of tolerance development (Al Nabhani and Eberl, 2020; Hornef and Torow, 2020). This establishes an intricate balance between the immune system and the microbiome.

In the gut, antibody–microbiota interactions take place at the mucosa (Neish, 2014). Under the mucosa, there is a single layer of enterocytes, which are specialised columnar epithelial cells, followed by the lamina propria (Kong et al., 2018). Secretory antibodies – predominantly sIgA, and to a lesser extent, sIgM, are also secreted into the intestinal mucosa and lumen (Janeway et al., 2001). Secretory antibodies are produced by plasma cells in the lamina propria of the gastrointestinal tract (GIT) (Janeway et al., 2001). Secretory Ig are able to interact with microbes at the mucosal surfaces in two ways – canonical binding within the Fragment antigen binding (Fab) region and non-canonical glycan-mediated binding via O-linked glycans on the hinge region, or N-linked glycans on the J chain and secretory component (Royle et al., 2003; Forbes et al., 2008; Mathias and Corthésy, 2011; Huus et al., 2021; Raskova Kafkova et al., 2021).

Specialised epithelial cells called microfold (M) cells take up antigens from the intestinal lumen and transport them across to antigen-presenting cells (dendritic cells and macrophages) and lymphocytes (T and B cells) at the basal surface (Janeway et al., 2001). In T cell-dependent class switching, antigen presentation results in plasma cells secreting IgA with high affinity and specificity to microbes (Huus et al., 2021). T cell-independent class switching takes place via BAFF (B cell-activating factor) and APRIL (a proliferation-inducing ligand) ligand binding to the TACI (transmembrane activator and calcium-modulator and cyclophilin ligand interactor) receptor on B cells, in the presence of retinoic acid, nitric oxide, and transforming growth factor β (TGFβ) (Huus et al., 2021). The T cell-independent pathway generally results in IgA with low affinity and specificity to microbiota (Huus et al., 2021). Both T cell-dependent and -independent pathways are required to maintain homeostasis with the microbiota (León and Francino, 2022; Gupta et al., 2023). The T cell-dependent pathway is thought to be largely in response to pathogens, while the T cell-independent pathway is in response to commensals, although the mechanisms are more complex and there is functional redundancy, with knocking out of both pathways being required to remove microbiota binding (Bunker et al., 2015; Gupta et al., 2023).

Binding of Ig to microbes in the GIT has classically been thought to be for immune exclusion, where binding to pathogens causes agglutination or aggregation for clearance via mucus entrapment and ciliary or peristaltic movement (Bunker and Bendelac, 2018; Hoces et al., 2020). This also prevents pathogens and pathobionts from binding to the epithelial layer and entering systemic circulation, while controlling the commensal population in the microbiota (Hoces et al., 2020). It has since been shown that Ig binding to microbes can also allow for immune inclusion, as in the case of the microbiota, where binding enables colonisation (Lingasamy et al., 2023). Up to 50% of the adult gut microbiota is bound by antibodies, and by day 10 of breastfeeding, up to 80% of bacteria in infant samples may be bound by antibodies (Gopalakrishna et al., 2019; Huus et al., 2021). Beyond immune exclusion or inclusion, binding of Ig to microbes has been shown to affect both Ig and microbes. The binding of Ig is able to influence gene expression and epitope production of the microbe, and microbes are also able to use the Ig as a carbon source for food (Planer et al., 2016; Briliūtė et al., 2019; Huus et al., 2020).

While much research has been conducted on the development of the infant gut microbiome in terms of factors influencing microbiome composition and changes in microbiota in relation to health outcomes, not much is known about the interactions between antibodies and the microbiota in development. As Ig–microbiota interactions are a function of the antibody, microbiota, and the environment, it is likely that the complexity in the changing environment, especially during development, also contributes to this.

Studies investigating Ig–microbiota interactions have used a technique we term Ig-Seq for standardisation, which incorporates fluorescence-activated cell sorting (FACS) or magnetic-activated cell sorting (MACS) of samples into fractions of microbiota bound or unbound by Ig, followed by sequencing of the fractions to determine differences in the composition of bacteria. However, methodological differences between studies have resulted in difficulty comparing the results and subsequently what is considered as the core Ig-bound microbiome.

As such, a scoping review was conducted to clarify and summarise what is known in the literature about Ig–microbiota interactions in the maternal and infant space (focusing on studies employing the Ig-Seq technique), detailing differences in studies and commonalities in bacteria bound or unbound to Ig across studies, as well as to identify knowledge gaps for further study. Given the limited number of studies, a scoping review was deemed to be more appropriate in mapping current evidence than a systematic review.

## Methods

### Search strategy

The scoping review was prepared using the Joanna Briggs Institute (JBI) Manual for Evidence Synthesis 2020 (Peters et al., 2020) and the Preferred Reporting Items for Systematic Reviews and Meta-Analysis extension for Scoping Reviews (PRISMA- ScR) with updated changes from the 2020 PRISMA statement (Tricco et al., 2018; Page et al., 2021).

A total of four databases were used to screen for articles regarding the Ig–bacteria interactions in breastmilk and stool of mothers and infants. The databases used were SCOPUS, MEDLINE, Embase, and Web of Science. A search was performed on 4th March 2024 using keywords and Medical Subject Headings (MeSH), combining the following concepts: microbiota AND immunoglobulins AND (gut OR stool OR breast milk) AND (maternal OR infants) AND (Ig-seq OR flow cytometry OR fluorescence activated cell sorting OR sequencing) (Supplementary Table S1). The Boolean operator “OR” was used instead of “AND” for the concepts flow cytometry/fluorescence activated cell sorting and sequencing due to the omission of relevant articles when using “AND.” The more sensitive approach using “OR” was hence chosen instead. The complete search strategy, including keywords, truncation, and Boolean operators for individual databases, is presented in Supplementary Table S2.

### Source of Evidence Screening and Selection

Retrieved results from the databases were exported into EndNote X9, and duplicate sources were removed. Remaining sources were then exported into Covidence for screening. Title and abstract screening, followed by full-text screening, were performed by two reviewers (ATYJ and CTC) according to inclusion and exclusion criteria. Inclusion criteria were (1) binding of Ig to microbiota, (2) in stool or breast milk samples, and (3) in infants or mothers. The exclusion criterion was Ig binding of a single bacterial taxon or cultured taxa instead of the microbiota in general, no sorting or sequencing, and no sequencing of fractions. Additional relevant articles were included through the search of reference lists, while sources that were not journal articles or that only included mice were excluded during full-text screening.

### Data extraction

The following information was extracted from the included studies:

- *Study characteristics* (author, year, country, aim, type of study, outcome, samples collected, number of timepoints, timepoints of collection of samples, Igs analysed, confounders, interventions, factors studied).
- *Participant characteristics* (groups compared, selection of participants, age in study, age at outcome, population).
- *Methodology* (DNA stain, cell sorting, controls, sequencing, Ig index used, analysis used for ASV transmission from maternal to infant samples).
- *Results* (Ig-Seq results, other findings, predominant or significant taxa in overall microbiota composition, predominant or significant taxa for composition of sorted Ig+ and Ig− fractions, ASVs transferred from maternal to infant samples as a measure of vertical transmission).

Authors were contacted to provide the required missing information where possible.

### Analysis and presentation of results

Ig-Seq data were grouped according to study aim, population, and analysis. Relevant parts of each study were then extracted based on the subsequent topics: (1) comparison of sorted fractions and the overall microbiota, (2) differences in binding of taxa according to type of antibody and characterisation of Ig–microbiota associations, (3) comparison of bacterial taxa bound (Ig+) and unbound (Ig−) to Ig in different sample types, (4) changes in Ig–microbiota binding patterns with time, and (5) Ig–microbiota binding patterns in health and disease. Healthy populations were used for analysis instead of populations with disorders for (1) to (4). The data were arranged according to sample type and Ig fraction, and recurrent taxa found across studies in each topic were then extracted where possible.

## Results

### Search results

**Figure 1.**
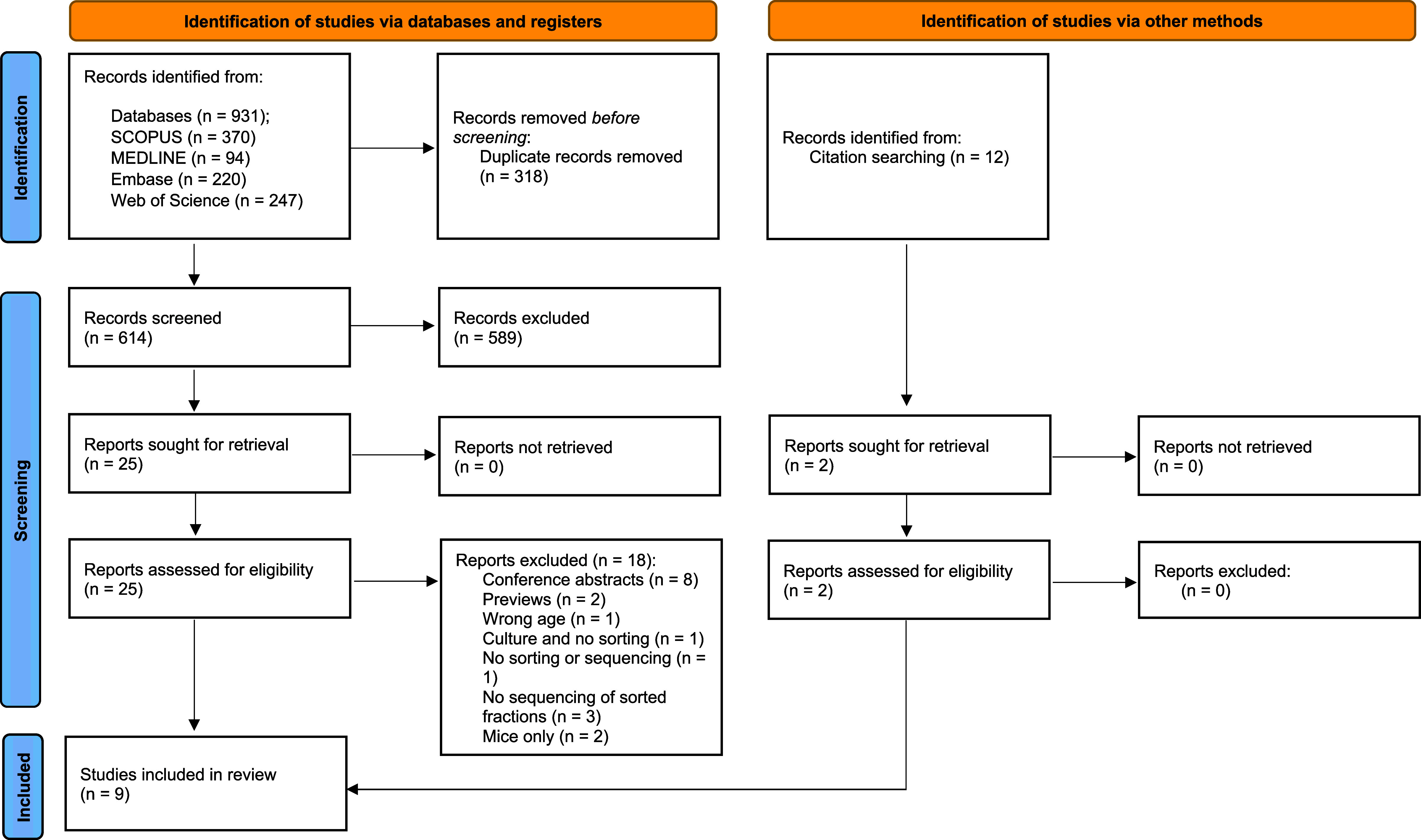
PRISMA 2020 flow diagram of identified, included, and excluded records. *From:* Page et al. (2021). Figure 1. long description.

A total of 931 sources were downloaded from databases (SCOPUS *n* = 370, MEDLINE *n* = 94, Embase *n* = 220, and Web of Science *n* = 247). After removal of duplicates, 614 sources were included for title and abstract screening. Due to the more sensitive approach used as described above, irrelevant articles not employing Ig-Seq were excluded based on exclusion criteria. A total of 25 sources were then assessed for eligibility, and eight conference abstracts and two previews were removed before full-text screening (Supplementary Table S3). From the remaining 15 journal articles, three articles were removed from full-text screening as they did not include sequencing of sorted fractions following flow cytometry, one article was removed as participants were of paediatric age (4 years of age) rather than infants, one only used cultured microbes and did not employ sorting, one article was removed for not having sorting or sequencing, and two were removed for only including mice. Hand searching from reference lists resulted in two additional articles that were included in the review (Figure 1). These articles did not include Ig-Seq, flow cytometry, sorting, or sequencing as keywords, while the methodology used employed cell sorting and sequencing, and were therefore included. This resulted in a total of nine articles included in the review at the end of the search (Supplementary Table S4).

### Characteristics of sources of evidence

General study characteristics, methodology, and findings of each source are presented in Supplementary Table S4. The compilation of predominant taxa or taxa found significant by statistical analysis in the overall microbiota or Ig+ or Ig− fractions was reported in Supplementary Table S5, while taxa of ASVs transmitted from maternal to infant samples in Qi et al. (2022) are detailed in Supplementary Table S6.

### Study and participant characteristics

The studies were published between 2015 and 2022 (Supplementary Table S4). Four studies were published within the past 5 years (Dzidic et al., 2020; Sánchez-Salguero et al., 2021;Ding et al., 2022; Qi et al., 2022), two studies were published in 2019 (Gopalakrishna et al., 2019; Janzon et al., 2019), and one study per year was published from 2015 to 2017 (Kau et al., 2015; Planer et al., 2016; Dzidic et al., 2017) (Supplementary Table S4). Six studies only involved humans (Dzidic et al., 2017; Janzon et al., 2019; Dzidic et al., 2020; Sánchez-Salguero et al., 2021; Ding et al., 2022; Qi et al., 2022) and three studies included both human and mouse models (Kau et al., 2015; Planer et al., 2016; Gopalakrishna et al., 2019) (Supplementary Table S4). The studies were conducted in Malawi (*n* = 1) (Kau et al., 2015), Mexico (*n* = 1) (Sánchez-Salguero et al., 2021), Sweden (*n* = 2) (Dzidic et al., 2017; Dzidic et al., 2020), China (*n* = 2) (Ding et al., 2022; Qi et al., 2022), and the USA (*n* = 3) (Planer et al., 2016; Gopalakrishna et al., 2019; Janzon et al., 2019) (Supplementary Table S4). All articles involved cohort studies (Kau et al., 2015; Planer et al., 2016; Dzidic et al., 2017; Gopalakrishna et al., 2019; Janzon et al., 2019;Dzidic et al., 2020; Sánchez-Salguero et al., 2021; Ding et al., 2022; Qi et al., 2022), with two of these articles involving two different cohorts within the study (Kau et al., 2015; Gopalakrishna et al., 2019) (Supplementary Table S4). The majority of studies were conducted on mother–infant pairs (*n* = 5) (Planer et al., 2016; Dzidic et al., 2020; Sánchez-Salguero et al., 2021; Ding et al., 2022; Qi et al., 2022), while four studies were only performed on infants (twins or singletons) (Kau et al., 2015; Dzidic et al., 2017;Gopalakrishna et al., 2019; Janzon et al., 2019) (Supplementary Table S4). The age of participants during the study ranged from birth to seven years of age (Supplementary Table S4). Most participant outcomes were measured in the first three years of life, although two studies measured outcomes up to seven years of age (Dzidic et al., 2017; Dzidic et al., 2020) (Supplementary Table S4).

### Aim and outcome measured

There were two main groups of studies – one group aimed to characterise Ig–microbiota associations (*n* = 5) (Planer et al., 2016; Janzon et al., 2019; Sánchez-Salguero et al., 2021;Ding et al., 2022; Qi et al., 2022), while the other group aimed to determine the role of Ig–microbiota associations in health and disease (*n* = 4) (Kau et al., 2015; Dzidic et al., 2017; Gopalakrishna et al., 2019; Dzidic et al., 2020). Characterisation of Ig–microbiota associations included understanding the antibody responses to gut microbiota development (IgA, IgG, and IgM) (Planer et al., 2016; Janzon et al., 2019), diversity and composition of Ig-coated and uncoated bacteria in maternal stool, breastmilk, and infant stool (Sánchez-Salguero et al., 2021; Ding et al., 2022), as well as vertical transmission of Ig-coated bacteria (Qi et al., 2022) (Supplementary Table S4). In terms of health and disease, the disorders studied were undernutrition-related enteropathy (*n* = 1) (Kau et al., 2015), allergic disease (*n* = 2) (Dzidic et al., 2017; Dzidic et al., 2020), and necrotising enterocolitis (NEC) (*n* = 1) (Gopalakrishna et al., 2019.

### Study design

The samples collected were maternal stool (*n* = 3) (Planer et al., 2016; Ding et al., 2022; Qi et al., 2022), breastmilk (*n* = 4, including 1 colostrum) (Dzidic et al., 2020; Sánchez-Salguero et al., 2021; Ding et al., 2022; Qi et al., 2022), and infant stool (*n* = 8, including 1 meconium) (Kau et al., 2015; Planer et al., 2016; Dzidic et al., 2017; Gopalakrishna et al., 2019; Janzon et al., 2019; Sánchez-Salguero et al., 2021; Ding et al., 2022; Qi et al., 2022) (Supplementary Table S4). The timepoint samples were collected at varied times according to study design, either pre- or post-partum (Planer et al., 2016; Sánchez-Salguero et al., 2021; Ding et al., 2022; Qi et al., 2022), intervention (Kau et al., 2015) or diagnosis (Kau et al., 2015; Dzidic et al., 2017; Gopalakrishna et al., 2019; Dzidic et al., 2020), or at regular timepoints across a period of development (Planer et al., 2016; Janzon et al., 2019) (Supplementary Table S4). One study collected and analysed samples according to milk stage (colostrum, transitional, and mature) (Ding et al., 2022) (Supplementary Table S4). Interventions used in the studies were diet (*n* = 1) (Kau et al., 2015) and probiotics (*n* = 2) (Dzidic et al., 2017; Dzidic et al., 2020), while the antibodies analysed were IgA (*n* = 6) (Kau et al., 2015; Planer et al., 2016; Dzidic et al., 2017; Gopalakrishna et al., 2019; Janzon et al., 2019; Dzidic et al., 2020), sIgA (*n* = 2) (Ding et al., 2022; Qi et al., 2022), IgA1 and IgA2 (*n* = 1) (Sánchez-Salguero et al., 2021), IgG (*n* = 2) (Gopalakrishna et al., 2019; Janzon et al., 2019), and IgM (*n* = 2) (Gopalakrishna et al., 2019; Janzon et al., 2019) (Supplementary Table S4).

### Ig-Seq

**Figure 2.**
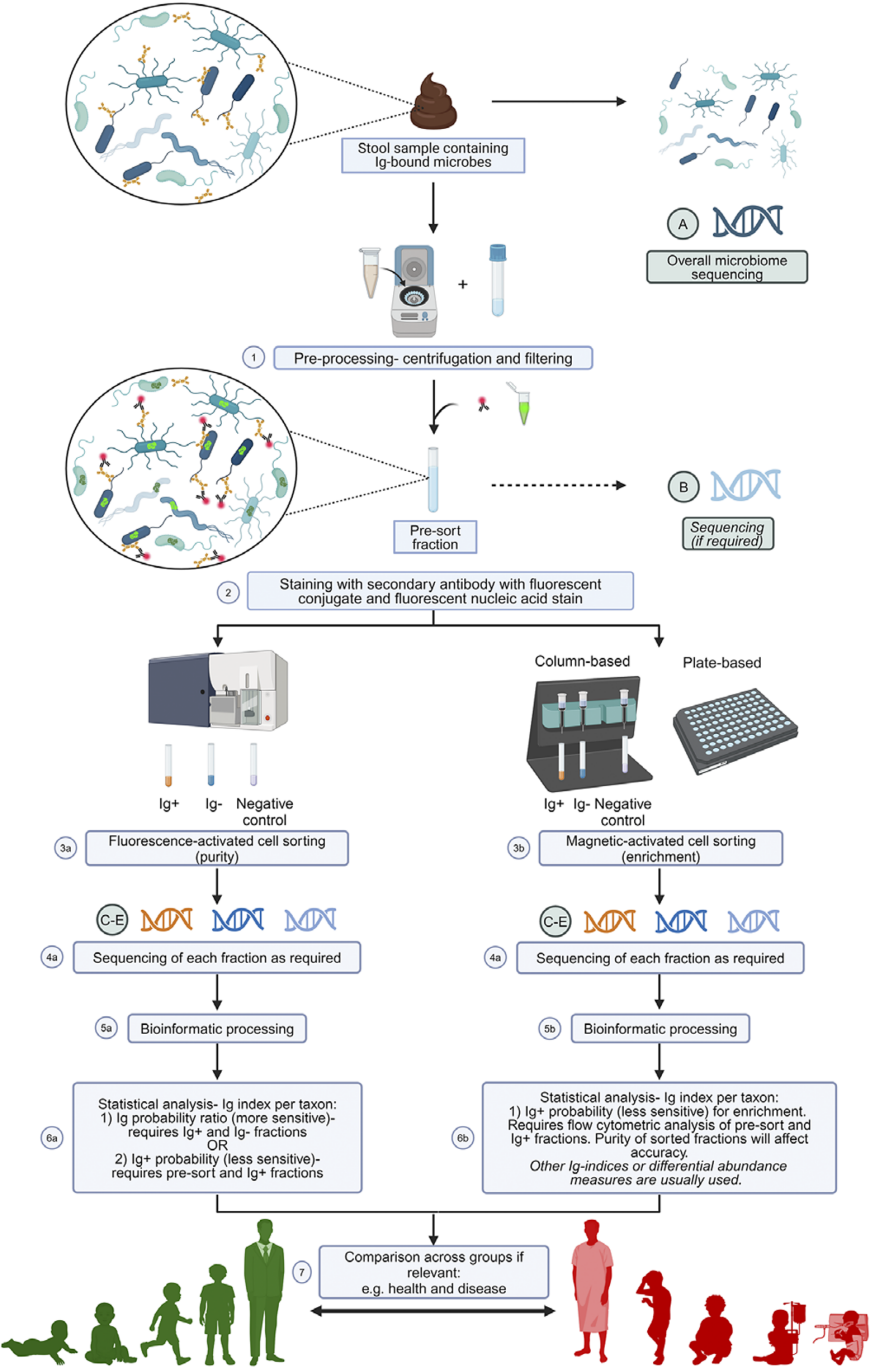
Suggested Ig-Seq workflow based on current literature. Ig-Seq starts from a sample containing Ig-bound microbes. No culturing of microbes from the sample or pre-mixing of serum with the sample (microbiota flow cytometry; mFLOW) is involved. Sequencing of the overall microbiome (A) should be performed. (1) Initial pre-processing of the sample for Ig-Seq with centrifugation and filtering is performed to remove debris. (2) This is followed by staining with a secondary antibody with a fluorescent conjugate and a fluorescent nucleic acid stain to obtain the pre-sort fraction. The pre-sort fraction (B) may also be sequenced. (3) Cell sorting of Ig-bound and -unbound microbiota can take place via fluorescence-activated cell sorting (FACS) to achieve greater purity (a), or magnetic-activated cell sorting (MACS) for enrichment of the Ig+ fraction (b). Both methods of sorting should include negative controls to account for contamination. MACS sorting has usually been performed using column-based sorting, as per papers in this review, while a newer plate-based MACS method has recently been proposed. (4) Sequencing of the Ig+ and Ig− fractions and negative controls (C-E) as required. Workflow from extraction to sequencing should be performed the same way as the overall microbiota to allow for comparison. (5) Bioinformatic processing of sequences to obtain microbiota composition, including decontamination and use of updated databases. (6a) Statistical analysis to obtain the Ig-index per taxon from FACS data, with promising approaches of either the Ig probability ratio for increased sensitivity, or the Ig+ probability ratio to only obtain taxa with greater binding affinity as a second option. The Ig probability ratio requires sequencing of Ig+ and Ig- fractions, while the Ig+ probability requires sequencing of the pre-sort and Ig+ fractions. (6b) The Ig+ probability may be used if flow cytometric analysis of the pre-sort and post-sort Ig+ fractions is performed. Purity of the sorted fractions will affect the results obtained. Other Ig-indices or differential abundance measures are usually used instead. (7) Comparison of Ig indices and microbiome between groups, if relevant, such as in health and disease. Created in BioRender.com and using Science Figures. Figure 2. long description.

A suggested workflow containing considerations for Ig-Seq based on current literature is depicted in Figure 2.

#### Terminology

Various terms were coined to describe the process of cell sorting antibody-bound and -unbound fractions, followed by sequencing of fractions – “BugFACS” (*n* = 2) (Kau et al., 2015; Planer et al., 2016), “IgSeq” (*n* = 1) (Gopalakrishna et al., 2019), and “IgA-Seq” (*n* = 1) (Sánchez-Salguero et al., 2021), while other studies did not use a specific term for combining both processes together and described them separately (*n* = 5) (Dzidic et al., 2017; Janzon et al., 2019; Dzidic et al., 2020; Ding et al., 2022; Qi et al., 2022) (Supplementary Table S4). This review will use the term “Ig-Seq” for consistency, which we propose for standardisation.

#### Cell sorting and controls

Cell sorting of samples into Ig+ or Ig− fractions was performed using either fluorescence-activated cell sorting (FACS) (*n* = 7) or magnetic-activated cell sorting (MACS) (*n* = 4) (Supplementary Table S4). A two-way sort into IgA+ and IgA− fractions was performed in seven studies (FACS *n* = 4 (Kau et al., 2015; Planer et al., 2016;Dzidic et al., 2017; Dzidic et al., 2020), MACS *n* = 2 (Gopalakrishna et al., 2019; Ding et al., 2022). One study did MACS sorting into IgA1+, IgA2+, and IgA− fractions (Sánchez-Salguero et al., 2021), and another study only used MACS to enrich the IgA+ population and did not collect the IgA− fraction (Qi et al., 2022). One study also performed a four-way sort into IgAM+, IgAMG+, IgA−, and IgG+ fractions (Janzon et al., 2019) (Supplementary Table S4). Two studies also mentioned analysing “input” (processed for FACS but unsorted) fractions (Kau et al., 2015; Planer et al., 2016) (Supplementary Table S4). DNA stains used for FACS sorting were SytoBC (*n* = 3) (Kau et al., 2015; Planer et al., 2016), SYTO62 (*n* = 2) (Dzidic et al., 2017; Dzidic et al., 2020), and Vybrant DyeCycle Violet (*n* = 1) (Janzon et al., 2019) (Supplementary Table S4). Controls included isotype, unstained and unsorted controls, verification of Ig staining specificity, and measures for contamination (sheath fluid autoclaved and replaced routinely, flow cytometer sterilisation, sequencing of sheath fluid samples pre and post sort, and removal of contaminants, and purging of the instrument between each sample) (Supplementary Table S4).

#### Sequencing, controls, and post-processing

Eight studies used bead beating in DNA extraction (Kau et al., 2015; Planer et al., 2016; Dzidic et al., 2017; Gopalakrishna et al., 2019; Dzidic et al., 2020; Sánchez-Salguero et al., 2021; Ding et al., 2022; Qi et al., 2022). Seven used kits for extraction (Dzidic et al., 2017; Gopalakrishna et al., 2019; Janzon et al., 2019; Dzidic et al., 2020; Sánchez-Salguero et al., 2021; Ding et al., 2022; Qi et al., 2022) and two used phenol–chloroform solutions for stool samples, but added cells from sorted fractions directly to PCR mixtures without extraction (Kau et al., 2015; Planer et al., 2016) (Supplementary Table S4). Additional enzymatic lysis (*n* = 2) (Dzidic et al., 2017; Dzidic et al., 2020) and alkali lysis for sorted cells (*n* = 1) (Janzon et al., 2019) were also used. Controls such as sampling material (*n* = 1) (Sánchez-Salguero et al., 2021), extraction blanks (*n* = 1) (Janzon et al., 2019), PCR negative controls (*n* = 3) (Planer et al., 2016; Janzon et al., 2019; Ding et al., 2022), and DNase in mastermix decontamination (*n* = 1) (Sánchez-Salguero et al., 2021) were mentioned (Supplementary Table S4). The majority of studies used short-read Illumina sequencing (*n* = 6) (Planer et al., 2016; Gopalakrishna et al., 2019; Janzon et al., 2019; Dzidic et al., 2020; Ding et al., 2022; Qi et al., 2022), while two studies used pyrosequencing (Kau et al., 2015; Dzidic et al., 2017) and one study used Ion Torrent sequencing (Sánchez-Salguero et al., 2021) of the 16S rRNA gene. Qi et al. (2022) also performed shotgun metagenomic sequencing on three out of 19 mother–infant samples that met the sequencing standard. Three studies used the V4 region of the 16S rRNA gene (Planer et al., 2016;Gopalakrishna et al., 2019; Janzon et al., 2019). Another three studies used the V3–4 region (Dzidic et al., 2020;Ding et al., 2022; Qi et al., 2022), and one study each used the V2 (Kau et al., 2015), V3 (Sánchez-Salguero et al., 2021), or V3–5 region (Dzidic et al., 2017 (Supplementary Table S4). Most studies used OTUs (operational taxonomic units) (*n* = 7) (Kau et al., 2015; Planer et al., 2016; Gopalakrishna et al., 2019; Janzon et al., 2019; Dzidic et al., 2020; Sánchez-Salguero et al., 2021; Ding et al., 2022), and the Greengenes database for classification (*n* = 3) (Janzon et al., 2019; Dzidic et al., 2020; Sánchez-Salguero et al., 2021), while Qi et al. (2022) used ASVs (amplicon sequence variants) with single nucleotide resolution (Supplementary Table S4). Two studies used a custom database based on a Greengenes “Isolated named strains 16S” database and modified phylogeny from the NCBI taxonomy database (Kau et al., 2015; Planer et al., 2016), one study used the NCBI 16S Microbial database (Qi et al., 2022) and one study used the SILVA database (Ding et al., 2022) (Supplementary Table S4). Filtering or removal of contaminants was mentioned in four studies (Kau et al., 2015; Planer et al., 2016; Dzidic et al., 2017; Dzidic et al., 2020), and one study used deconvolution to decrease the effect of contamination in Ig-Seq (Gopalakrishna et al., 2019 (Supplementary Table S4).

### Ig index and other methods of analysis

Studies varied in the methods used for analysis of Ig-Seq data (Supplementary Table S4). Two articles used the IgA index described by Kau et al. (2015) as a measure of overall binding of a taxon in the IgA+ or IgA− fractions (Kau et al., 2015; Planer et al., 2016) (Supplementary Table S4). Dzidic et al. (2017) and Dzidic et al. (2020) used the ratio of relative abundance proportions between the IgA+ and IgA− fractions (log (IgA+/IgA−)) as a degree of mucosal responsiveness (Supplementary Table S4). Further statistical analysis was then performed on IgA indices to determine significance (paired Wilcoxon test (Kau et al., 2015), Wilcoxon signed rank test (Planer et al., 2016), linear discriminant analysis (LDA) effect size (LEfSe) analysis to compare IgA indices between groups (Dzidic et al., 2017) (Supplementary Table S4). Janzon et al. (2019) used a linear mixed model to look for common OTUs differentially abundant between FACS quadrants instead, and a weighted transfer ratio (WTR) was used as a measure of transfer of a species between sample types in Qi et al. (2022) (Supplementary Table S4). Other studies reported differentially abundant taxa between groups using LEfSe (Gopalakrishna et al., 2019; Sánchez-Salguero et al., 2021) or compared the relative abundance of the predominant taxa (Ding et al., 2022) (Supplementary Table S4). Planer et al. (2016) and Qi et al. (2022) also provided an additional indicator species analysis and core microbiome analysis respectively (Supplementary Table S4).

### Comparison of bacteria in sorted fractions to the overall microbiota

The composition of bacteria in sorted fractions of the overall unsorted microbiota differed according to the study (Supplementary Table S7). Out of the five studies compared (Gopalakrishna et al., 2019; Janzon et al., 2019; Sánchez-Salguero et al., 2021; Ding et al., 2022; Qi et al., 2022), the majority of the studies showed some overlap between the sorted fractions and overall microbiota. In Gopalakrishna et al. (2019), the overall microbiota of stool of healthy infants was associated with *Lachnospiraceae* according to LDA analysis, while the sorted fraction had an increased relative abundance of Clostridiales and Bifidobacteriales and decreased relative abundance of *Enterobacteriaceae*. Janzon et al. (2019) used linear mixed models to identify taxa differentially abundant between sorted fractions and overall microbiota in infant stool, with Bacteroidetes, Verrucomicrobia, Gammaproteobacteria, and Firmicutes enriched in the overall microbiota, as compared to Actinobacteria and Alphaproteobacteria being enriched in the sorted fraction. Sánchez-Salguero et al. (2021) compared IgA2-associated taxa and the overall microbiota in neonatal stool using LEfSe analysis. Bacteria in the overall microbiota included *Caulobacteraceae* and *Sanguibacter*, while IgA2-associated bacteria included Alphaproteobacteria, *Pseudomonas*, and *Bifidobacterium*. In breastmilk, the overall microbiota included *Clostridium*, *Streptococcus*, *Pseudomonas*, *Bacteroides*, *Bifidobacterium*, and *Corynebacterium*, while IgA2-associated taxa included *Bacteroides*, *Bifidobacterium*, and *Lactobacillus*, using relative abundance (Sánchez-Salguero et al., 2021). There was some overlap between fractions in Ding et al. (2022) according to the relative abundance of predominant taxa. In infant stool, *Bifidobacterium*, *Streptococcus*, *Escherichia-Shigella*, and *Bacteroides* were found in both sorted fractions and overall microbiota. In maternal stool, *Bifidobacterium*, *Bacteroides*, *Blautia*, *Faecalibacterium*, and *Escherichia-Shigella* were common to both sorted fractions and overall microbiota, while in breastmilk, common taxa were *Bifidobacterium*, *Pseudomonas*, and *Escherichia-Shigella* (Ding et al., 2022). In Qi et al. (2022), taxa in the top 30 species significantly enriched in each sample type according to Kruskal–Wallis tests were reported. There were no shared taxa in infant stool between the sorted fractions and the overall microbiota, while *Blautia wexlerae* was shared between sorted fractions and overall microbiota in maternal stool, and *Staphylococcus capitis* was common to both fractions in breastmilk (Qi et al., 2022).

#### Recurrent bacteria across studies

In infant stool (*n* = 5), recurrent bacteria found in the overall microbiota across studies at the family level were *Lachnospiraceae* (*n* = 2) (Gopalakrishna et al., 2019; Janzon et al., 2019) and *Enterobacteriaceae* (*n* = 2) (Janzon et al., 2019; Ding et al., 2022), *Bacteroides*, *Lactobacillus*, *Veillonella* (Janzon et al., 2019; Ding et al., 2022), and *Bifidobacterium* at genus level (*n* = 2) (Ding et al., 2022; Qi et al., 2022) (Table 1). Recurrent sorted bacteria were Bifidobacteriales, *Bifidobacterium* (*n* = 4) (Gopalakrishna et al., 2019; Janzon et al., 2019; Sánchez-Salguero et al., 2021; Ding et al., 2022), *Paracoccus* (*n* = 2) (Janzon et al., 2019; Sánchez-Salguero et al., 2021), and *Pseudomonas* (*n* = 3) (Janzon et al., 2019; Sánchez-Salguero et al., 2021; Ding et al., 2022). In maternal stool (*n* = 2), Ding et al. (2022) and Qi et al. (2022) found *Blautia* and *Faecalibacterium* in the overall microbiota, and *Blautia, Bifidobacterium, Faecalibacterium,* and *Escherichia* in the sorted fraction (Ding et al., 2022; Qi et al., 2022). In breastmilk (*n* = 3), Sánchez-Salguero et al. (2021) and Ding et al. (2022) reported *Bifidobacterium, Pseudomonas,* and *Streptococcus* in the overall microbiota, and *Bifidobacterium* in the sorted fraction. *Staphylococcus* was also reported in two studies in the overall microbiota (Ding et al., 2022; Qi et al., 2022).

**Table 1.** Recurrent sorted and unsorted (overall microbiota) taxa according to sample type Table 1. long description.

Sample	Recurrent unsorted bacteria (overall microbiota)				Recurrent sorted (Ig+ and Ig− combined) bacteria			
	Order	Family	Genus	Study	Order	Family	Genus	Study
Infant stool		f_Lachnospiraceae		Gopalakrishna et al., 2019; Janzon et al., 2019	o_Bifidobacteriales		g_Bifidobacterium	Gopalakrishna et al., 2019; Janzon et al., 2019; Sánchez-Salguero et al., 2021; *Ding et al., 2022*
		f_Enterobacteriaceae		Janzon et al., 2019; *Ding et al., 2022*			g_Paracoccus	Janzon et al., 2019; Sánchez-Salguero et al., 2021
			g_Bacteroides	Janzon et al., 2019; *Ding et al., 2022*			g_Pseudomonas	Janzon et al., 2019; Sánchez-Salguero et al., 2021; *Ding et al., 2022*
			g_Lactobacillus	Janzon et al., 2019; *Ding et al., 2022*				
			g_Veillonella	Janzon et al., 2019; *Ding et al., 2022*				
			g_Bifidobacterium	*Ding et al., 2022*; *Qi et al., 2022*				
Maternal stool			g_Blautia	*Ding et al., 2022*; *Qi et al., 2022*			g_Blautia	*Ding et al., 2022*; *Qi et al., 2022*
			g_Faecalibacterium	*Ding et al., 2022*; *Qi et al., 2022*			g_Bifidobacterium	*Ding et al., 2022*; *Qi et al., 2022*
							g_Faecalibacterium	*Ding et al., 2022*; *Qi et al., 2022*
							g_Escherichia	*Ding et al., 2022*; *Qi et al., 2022*
Breastmilk			g_Bifidobacterium	*Sánchez-Salguero et al., 2021*; *Ding et al., 2022*			g_Bifidobacterium	*Sánchez-Salguero et al., 2021*; *Ding et al., 2022*
			g_Pseudomonas	*Sánchez-Salguero et al., 2021*; *Ding et al., 2022*				
			g_Streptococcus	*Sánchez-Salguero et al., 2021*; *Ding et al., 2022*				
			g_Staphylococcus	*Ding et al., 2022*; *Qi et al., 2022*				

*Note:* A total of *n* = 5 studies for infant stool, *n* = 2 studies for maternal stool, and *n* = 3 studies for breastmilk were used. The lowest common taxonomic level of bacterial taxa observed in at least two studies was recorded. *Italics* indicate study results that were based on relative abundance without differential analysis. We caveat that these findings are study-specific with limited comparability.

### Comparison of bacteria bound by type of Ig and characterisation of Ig–microbiota associations

Three studies investigated differences in the binding of microbes by different antibodies. Gopalakrishna et al. (2019) reported that IgA bound a greater percentage of microbes in infant stool than IgM or IgG based on flow cytometry. Most formula-fed infants (*n* = 11/19) had <1% of IgA-bound bacteria as expected with formula-feeding, although some infants had up to ~40% of IgA-bound bacteria that could not be explained (Gopalakrishna et al., 2019). Janzon et al. (2019) compared the binding patterns of bacteria in infant stool by IgA, IgM, and IgG using IgAM+, IgAMG+, and IgG+ sorted fractions. There was similar Ig binding across fractions, with Actinobacteria and Firmicutes being bound at the phylum level, and *Bifidobacteriaceae* being bound at the family level. While there was a large overlap of bacteria bound by all three antibodies, it was concluded that IgA and IgM were found to bind similar microbes, while IgG bound a different subset of microbes (Janzon et al., 2019). Sánchez-Salguero et al. (2021) compared the IgA1 and IgA2 fractions in colostrum using MACS sorting and LEfSe analysis. *Staphylococcus, Streptococcus,* and *Clostridium* were preferentially bound by IgA1, whereas *Bifidobacterium, Lactobacillus,* and *Bacteroides,* were preferentially bound by IgA2 (Sánchez-Salguero et al., 2021), with different groups of bacteria being recognised by the IgA isotypes. No IgA1+ bacteria were found in neonatal stool samples. IgA2+ bacteria in neonatal stool may have originated from colostrum samples based on a Source Tracker analysis, with a possibility of colostrum as a source of microbes and a role of IgA2-association of microbiota in infant gut development.

### Comparison of Ig-bound and -unbound bacteria in different sample types

Eight studies were included for infant stool and three studies for maternal stool and breastmilk (Supplementary Table S8). In infant stool, *Bifidobacteriaceae* (*n* = 3–4) (Planer et al., 2016; Janzon et al., 2019; Sánchez-Salguero et al., 2021; Ding et al., 2022), *Streptococcaceae* (*n* = 2–3) (Planer et al., 2016; Dzidic et al., 2017; Ding et al., 2022), and *Bifidobacterium* (*n* = 3) (Planer et al., 2016; Sánchez-Salguero et al., 2021; Ding et al., 2022) were found in both Ig+ and Ig− fractions. *Enterobacteriaceae* (*n* = 2) (Kau et al., 2015; Ding et al., 2022) was also bound to Ig, while *Lachnospiraceae* (*n* = 2) (Planer et al., 2016; Janzon et al., 2019) was not bound to Ig. Overlap of taxa found in both the Ig+ and Ig− fractions was observed when measures of relative abundance were used without differential analysis (Dzidic et al., 2017; Janzon et al., 2019; Ding et al., 2022). There was no overlap in taxa between Ig+ and Ig− fractions when IgA indices (Kau et al., 2015; Planer et al., 2016) or linear mixed models (Janzon et al., 2019) were used. In maternal stool, *Escherichia, Bifidobacterium* (*n* = 3) (Planer et al., 2016; Ding et al., 2022; Qi et al., 2022) and *Blautia* (*n* = 2) (Ding et al., 2022; Qi et al., 2022) were frequently associated with Ig and *Bacteroides* (*n* = 2) (Kau et al., 2015; Planer et al., 2016) was not associated with Ig. Bacteria that were Ig-associated in breastmilk were *Staphylococcus, Streptococcus* (*n* = 3) (Sánchez-Salguero et al., 2021; Ding et al., 2022; Qi et al., 2022)*, Bifidobacterium*, and *Pseudomonas* (*n* = 2) (Sánchez-Salguero et al., 2021; Ding et al., 2022). Recurrent taxa in the Ig− fraction could not be compared due to insufficient studies. Across sample types, *Bifidobacterium* was the only taxon that was found to be Ig-associated in infant stool, maternal stool, and breastmilk (Table 2). No recurrent taxa were seen across sample types in the Ig− fraction.

**Table 2.** Recurrent Ig-responses to microbiota according to sample type Table 2. long description.

Sample	Recurrent Ig+ bacteria				Recurrent Ig− bacteria			
	Order	Family	Genus	Study	Order	Family	Genus	Study
Infant stool		f_Bifidobacteriaceae	g_Bifidobacterium	Planer et al., 2016; *Janzon et al., 2019*; Sánchez-Salguero et al., 2021; *Ding et al., 2022*		f_Bifidobacteriaceae	g_Bifidobacterium	Planer et al., 2016; *Janzon et al., 2019*; *Ding et al., 2022*
		f_Streptococcaceae		*Dzidic et al., 2017*; *Ding et al., 2022*		f_Streptococcaceae		Planer et al., 2016; *Dzidic et al., 2017*; *Ding et al., 2022*
		f_Enterobacteriaceae		Kau et al., 2015; *Ding et al., 2022*		f_Lachnospiraceae		Planer et al., 2016; *Janzon et al., 2019*
Maternal stool			g_Escherichia	Planer et al., 2016; *Ding et al., 2022*; *Qi et al., 2022*			g_Bacteroides	Kau et al., 2015; Planer et al., 2016
			g_Bifidobacterium	Planer et al., 2016; *Ding et al., 2022; Qi et al., 2022*				
			g_Blautia	*Ding et al., 2022*; *Qi et al., 2022*				
Breastmilk			g_Staphylococcus	Sánchez-Salguero et al., 2021; *Ding et al., 2022*; *Qi et al., 2022*	Insufficient studies			
			g_Streptococcus	Sánchez-Salguero et al., 2021; *Ding et al., 2022*; *Qi et al., 2022*				
			g_Bifidobacterium	Sánchez-Salguero et al., 2021; *Ding et al., 2022*				
			g_Pseudomonas	Sánchez-Salguero et al., 2021; *Ding et al., 2022*				

*Note:* A total of *n* = 8 studies for infant stool and *n* = 3 studies for maternal stool and breastmilk. For Ig− taxa, breastmilk was not recorded due to insufficient studies. The lowest common taxonomic level of bacterial taxa observed in at least two studies was recorded. *Italics* indicate study results that were based on relative abundance without differential analysis. We caveat that these findings are study-specific with limited comparability.

Qi et al. (2022) used a WTR to compare transmission of ASVs between sample pairs as a measure of vertical transmission (Supplementary Table S6). IgA-associated lactic acid bacteria such as *Lactobacillus gasseri,* *Enterococcus faecium,* and *Streptococcus salivarius* were common in both breastmilk and infant stool samples (Qi et al., 2022). *Bifidobacterium longum* was the predominant bacterium transferred from the maternal gut to breastmilk. Strains shared between breastmilk and infant stool in three mother–infant pairs were also determined using shotgun-sequencing metagenomics and a database based on the WTR results (Supplementary Table S6) (Qi et al., 2022). *B. longum* subsp. *infantis* BT1 (*n* = 3 mother–infant pairs), *B. longum* subsp. *infantis* JCM 11347 (*n* = 2), and *L. gasseri* HL75 (1 mother–infant pair) were found to be common and potentially transmitted from mother to infant (Qi et al., 2022).

### Changes in Ig–microbiota binding patterns with time

Three studies looked at changes in Ig–microbiota binding patterns over time during infant gut development (Supplementary Table S9). Planer et al. (2016) reported that IgA–microbiota binding patterns were more similar in twin pairs up to 21 months of age and more dissimilar at 2 years of age. In unrelated infants, Ig–microbiota binding patterns were weakly correlated at 6 months of age and became more correlated at 2 years of age, when IgA responses resembled those of their adult mothers (Planer et al., 2016). Thirty OTUs that were consistently bound or not bound by IgA after 3 months of age were also identified, including *Akkermansia municiphila, Ruminococcus, Clostridium,* and *Bifidobacterium bifidum* in the IgA+ fraction, and *Roseburia, B. longum, Streptococcus, Bacteroides, Clostridium, Peptoclostridium, Blautia,* and *Ruminococcus* in the IgA− fraction (Planer et al., 2016). Janzon et al. (2019) discovered 2 OTUs in their study from Planer et al. (2016) that were bound (*Bifidobacterium*) or not bound (*Peptostreptococcaceae*) as previously described. Ding et al. (2022) investigated the changes in sample types according to milk stages (colostrum, transitional, and mature). In infant stool, breastmilk, and maternal stool, *Escherichia-Shigella* was the predominant IgA-associated bacteria, and *Pseudomonas* was the predominant non-IgA-associated bacteria at all stages. Planer et al. (2016) analysed the Ig–microbiota binding patterns in maternal stool at the peripartum stage, 6 months postpartum, and more than 12 months postpartum. The predominant IgA-associated taxon was *Ruminococcus torques* after 12 months postpartum.

#### Recurrent bacteria across studies at shared timepoints

Common timepoints for sample collection across studies were determined and used for analysis. Ig–microbiota binding patterns at common timepoints across all studies were analysed, with four timepoints for infant stool, one timepoint for maternal stool, and two timepoints for breastmilk (Supplementary Table S10 and Table 3). *Bifidobacterium, Escherichia,* and *Pseudomonas* (*n* = 2) (Sánchez-Salguero et al., 2021; Ding et al., 2022) were recurrent IgA-associated bacteria at 1 week of age in infant stool, *Ruminococcaceae* (*n* = 2) (Planer et al., 2016; Dzidic et al., 2017) and *Bifidobacteriaceae* (*n* = 2) (Planer et al., 2016; Janzon et al., 2019) at 12 months of age, and *Bifidobacteriaceae* (*n* = 2) (Planer et al., 2016; Janzon et al., 2019) at 16 months of age. No recurrent Ig-associated taxa were seen at one month of age (*n* = 2) (Ding et al., 2022; Dzidic et al., 2017). For the Ig− fraction, Clostridiales (*n* = 2) (Planer et al., 2016; Gopalakrishna et al., 2019) and Bifidobacteriales (*n* = 2) (Ding et al., 2022; Gopalakrishna et al., 2019) were recurrent at 1 month of age, *Bacteroidaceae, Ruminococcaceae, Streptococcaceae* (*n* = 2) (Planer et al., 2016; Dzidic et al., 2017), and *Lachnospiraceae* (*n* = 3) (Planer et al., 2016; Dzidic et al., 2017; Janzon et al., 2019) were recurrent at 12 months of age, and *Lachnospiraceae* (*n* = 2) (Planer et al., 2016; Janzon et al., 2019) was recurrent at 16 months of age. There were insufficient studies to compare IgA− taxa at 1 week of age in infant stool, maternal stool, and breastmilk. *Bifidobacterium, Escherichia,* and *Blautia* (*n* = 2) (Ding et al., 2022; Qi et al., 2022) were shared IgA-associated taxa at 1 month postpartum in maternal stool, while *Bifidobacterium* (*n* = 2) (Sánchez-Salguero et al., 2021; Ding et al., 2022) was shared in breastmilk at the colostrum stage at 1 week postpartum. There were no recurrent IgA-associated taxa at 1 month postpartum in the mature milk stage (*n* = 2) (Ding et al., 2022; Qi et al., 2022).

**Table 3.** Recurrent Ig+ and Ig− bacteria across time in infant gut development (infant stool), maternal stool, and breastmilk Table 3. long description.

Sample type	Timepoint	Recurrent Ig+ bacteria				Recurrent Ig− bacteria			
		Order	Family	Genus	Study	Order	Family	Genus	Study
Infant stool	1 week of age			g_Bifidobacterium	Sánchez-Salguero et al., 2021; *Ding et al., 2022*	Insufficient studies			
				g_Escherichia	Sánchez-Salguero et al., 2021; *Ding et al., 2022*				
				g_Pseudomonas	Sánchez-Salguero et al., 2021; *Ding et al., 2022*				
	1 month of age	No recurring taxa			*Ding et al., 2022*; Dzidic et al., 2017	o_Clostridiales			Planer et al., 2016; Gopalakrishna et al., 2019
						o_Bifidobacteriales			Gopalakrishna et al., 2019; *Ding et al., 2022*
	12 months of age		f_Ruminococcaceae		Planer et al., 2016; *Dzidic et al., 2017*		f_Bacteroidaceae		Planer et al., 2016; *Dzidic et al., 2017*
			f_Bifidobacteriaceae		Planer et al., 2016; *Janzon et al., 2019*		f_Ruminococcaceae		*Planer et al., 2016*; Dzidic et al., 2017
							f_Streptococcaceae		Planer et al., 2016; *Dzidic et al., 2017*
							f_Lachnospiraceae		Planer et al., 2016; *Dzidic et al., 2017*; Janzon et al., 2019
	16 months of age		f_Bifidobacteriaceae		Planer et al., 2016; *Janzon et al., 2019*		f_Lachnospiraceae		Planer et al., 2016; *Janzon et al., 2019*
Maternal stool	1 month postpartum			g_Bifidobacterium	*Ding et al., 2022*; *Qi et al., 2022*	Insufficient studies			
				g_Blautia	*Ding et al., 2022*; *Qi et al., 2022*				
				g_Escherichia	*Ding et al., 2022*; *Qi et al., 2022*				
Breastmilk	1 week postpartum (colostrum stage)			g_Bifidobacterium	Sánchez-Salguero et al., 2021; *Ding et al., 2022*	Insufficient studies			
	1 month postpartum (mature milk stage)	No recurring taxa			*Ding et al., 2022*; *Qi et al., 2022*				

*Note:* For Ig+ bacteria, a total of *n* = 2 studies for 1 week, 1 month, and 16 months of age, *n* = 3 studies for 12 months of age were used for infant stool, and *n* = 2 studies were used for maternal stool and breastmilk. For Ig− bacteria, in infant stool, *n* = 2 studies were used for 1 month, *n* = 4 for 12 months of age, and *n* = 3 studies for 16 months of age. Timepoint at 1 week of age for infant stool was omitted due to insufficient studies (*n* = 1), as were maternal stool and breastmilk. The lowest common taxonomic level of bacterial taxa observed in at least two studies was recorded. *Italics* indicate study results that were based on relative abundance without differential analysis. We caveat that these findings are study-specific with limited comparability.

### Ig–microbiota binding patterns in health and disease

Differences in Ig-bacterial microbiota binding patterns in health and disease were investigated in four studies.

#### Undernutrition (n = 1)

Kau et al. (2015) focused on the IgA+ fraction in undernutrition and kwashiorkor development in humans and used humanised mice. In GF mice transplanted with stool from a 21-month-old twin pair discordant for kwashiorkor, *Verrucomicrobiaceae* was enriched in the IgA+ fraction in the healthy microbiota group. *Enterobacteriaceae* were enriched in the IgA+ fraction in mice harbouring kwashiorkor twin pair microbiota and fed an undernutritious Malawian diet (Kau et al., 2015). Diet also influenced IgA–microbiota binding patterns, with *Erysipelotrichaceae* being IgA-associated in mice fed the Malawian diet but not in mice fed standard diets (Kau et al., 2015). In a Malawian twin cohort of kwashiorkor discordant twin pairs and healthy concordant twin pairs, discordant twins with kwashiorkor had significantly enriched *Enterobacteriaceae* in the IgA+ fraction as compared to healthy concordant twins at the time of diagnosis. A peanut-based ready-to-use-therapeutic food (RUTF) therapy for 4 weeks was able to decrease IgA-associated *Enterobacteriaceae* significantly due to a diet with adequate nutrients (Kau et al., 2015).

#### Allergic disease (n = 2)

Dzidic et al. (2017) investigated IgA-bound microbiota in stool collected at 1 and 12 months of age in allergic disease and healthy children at 7 years of age. At 1 month of age, *Clostridium sensu stricto* was enriched in the IgA+ fraction of children with allergy, and Acinetobacter was enriched in children with asthma compared to children remaining healthy (Dzidic et al., 2017). In the IgA− fraction, *Parabacteroides, Faecalibacterium,* and *Anaerococcus* were enriched in children with allergy, *Faecalibacterium* was enriched in children with asthma, and *Micrococcus* was enriched in children who developed allergic rhinoconjunctivitis (ARC) as compared with healthy children (Dzidic et al., 2017). At 12 months of age, *Lachnospiraceae inc. sed*. was enriched in the IgA+ fraction in children with allergy, while *Roseburia* and *Erysipelotrichaceae inc. sed*. were enriched in healthy children (Dzidic et al., 2017). In the IgA− fraction, *Bacteroides* was enriched in children with allergy, while healthy children had enriched *Veillonella* and *Delftia* (Dzidic et al., 2017). Children with asthma had enriched *Escherichia/Shigella* and children with ARC had enriched *Rhodanobacter* and *Bacteroides* in the IgA− fraction (Dzidic et al., 2017). In a later study, Dzidic et al. (2020) analysed the IgA-binding patterns in breastmilk 1 month postpartum and allergy development of children. However, no differences in IgA binding patterns or proportions of IgA-bacteria associations were found between mothers whose children did or did not develop allergy or asthma, and the authors concluded that decreased bacterial richness at 1 month of age was associated with allergy development (Dzidic et al., 2020). Probiotic treatment was able to influence the overall breastmilk microbiota composition, but no significant differences were found in Ig–microbiota binding patterns (Dzidic et al., 2020).

#### Necrotising enterocolitis (n = 1)

Differences in maternal IgA binding of microbiota in preterm infants and necrotising enterocolitis (NEC) development were explored by Gopalakrishna et al. (2019). In infant stool collected at 7–40 days of age, the overall microbiota was dominated by *Enterobacteriaceae* in infants that developed NEC, while infants who remained healthy had microbiota predominant in *Lachnospiraceae* (Gopalakrishna et al., 2019). Infants who developed NEC lacked IgA-bound *Enterobacteriaceae*, while healthy infants had decreased relative abundance of *Enterobacteriaceae* and increased relative abundance of anaerobes Clostridiales and Bifidobacteriales bound with IgA (Gopalakrishna et al., 2019).

## Discussion

While Ig–microbiota interactions are important, little is known about Ig–microbiota binding patterns during development and how they change with time. Studies have employed the technique Ig-Seq to study Ig–microbiota binding patterns, but methodological differences have made it difficult to define a core Ig-bound microbiome. This scoping review was conducted with the aim of presenting current literature of Ig–microbiota interactions in the maternal and infant space, to describe experimental differences between studies, define what bacteria were found Ig-bound and unbound across studies, suggest a standardised workflow for Ig-Seq and to identify areas for future study.

## Methodology

### Terminology

There is currently a lack of standardisation for the technique involving FACS or MACS sorting of bacteria into Ig-bound or unbound fractions, followed by sequencing of fractions. While various terms have been given to describe this, or merely describing it as sorting followed by sequencing of fractions, we propose the use of the term “Ig-Seq” for consistency. We would like to caveat that Ig-Seq is conceptually different from the technique mFLOW (microbiota flow cytometry) found in the literature and the use of the terms should be distinguished (detailed in Supplementary Text).

### Cell sorting and controls

A variety of nucleic acid dyes were used to stain bacteria. Some considerations would be the ability to stain both gram-positive and gram-negative bacteria, dye permeability, and lack of spectral overlap with the fluorescent dye used in the secondary antibody.

Choosing of the antibody isotype and type of sort is dependent on the research study. Most studies employed a two-way sort for IgA, as sIgA is the dominant antibody in both stool and breastmilk and most commonly associated with commensals (Pabst and Slack, 2020). Other studies looked at the difference or overlap in binding across Ig isotypes using a four-way sort. The results will be discussed below. IgA is the most important isotype to consider in Ig-Seq involving stool and breastmilk.

Cell sorting has been performed using both MACS and FACS. FACS will give fractions with greater purity than MACS, whereas MACS will focus on enrichment rather than pure fractions of bacteria bound and unbound to Ig (Figure 2). This was demonstrated by Qi et al. (2022), where only the Ig+ fraction was collected, but not the Ig− fraction. However, some papers using MACS were able to assess for purity using flow cytometry or performed deconvolution to control for contamination in the sort (Gopalakrishna et al., 2019; Sánchez-Salguero et al., 2021). These processes can be considered if intending to use MACS rather than FACS due to a lack of access to equipment or cost constraints. Some controls used during FACS include collecting sheath fluid from the fluidics pre and post sorting (Kau et al., 2015; Planer et al., 2016), and ensuring that backflush was performed between samples to maintain baseline background (Janzon et al., 2019). One study mentioned autoclaving of sheath fluid (Kau et al., 2015). However, autoclaving will introduce molecular contamination (Suyama and Kawaharasaki, 2013) while removing microbial contamination, and controls for molecular contamination should be used in conjunction to allow for filtering of contaminant sequences. Some studies also sequenced the input fraction that was processed for sorting but not put through the cell sorter as a baseline comparison post-sorting (Kau et al., 2015; Planer et al., 2016). This could also serve as a control for sequence contamination from the sorting process. Recently, a new method using 96-well plate-based MACS sorting (next-generation IgA-SEQ) has been proposed, as opposed to column-based MACS sorting (van Gogh et al., 2024). Briefly, the authors suggest that this improves yield for downstream metagenomics to be possible, maintains anaerobic conditions, and is less time-consuming compared to FACS (van Gogh et al., 2024). The authors also do not suggest column-based sorting in high microbiota abundance sample types due to problems in enrichment, and results may not accurately reflect real sample data (van Gogh et al., 2024). Because of previous findings that species-level IgA-coating between FACS and plate-based MACS sorting was only consistent in 60% of cases (van Gogh et al., 2024), interpretation of results in future studies will need to consider differences in methodology used. Comparison of Ig-unbound bacteria between plate-based MACS and FACS was not mentioned either (van Gogh et al., 2024), and this may need to be explored if using the Ig probability ratio discussed below.

### Sequencing, controls, and post-processing

The majority of studies used extraction kits for DNA extraction, including bead-beating for cell lysis. Bead-beating is able to improve DNA yield and affects genus characterisation, and should be incorporated in the extraction (Zhang et al., 2021). We recommend using extraction kits over manual phenol–chloroform extraction for greater consistency in results and in the consistency in purity of DNA extracted based on A260/280 absorbance (Douglas et al., 2020), and to also be consistent in the extraction procedure if also extracting overall sample material for general microbiome analysis (non-Ig-Seq) to allow for comparison. Controls, including extraction blanks, PCR negative controls, positive controls, and use of master mixes should also be detailed as per the Strengthening The Organization and Reporting of Microbiome Studies (STORMS) checklist, in line with current microbiome guidelines (Mirzayi et al., 2021). The majority of studies used Illumina sequencing, and only one study also included metagenomic sequencing. However, the number of samples that met the metagenomic sequencing standard was low (3 out of 19 mother–infant pairs). The majority of studies also used the regions within the V3–V5 area of the 16S rRNA gene (the most common V4), except for one study. Differences in composition based on the sequencing of different hypervariable regions of the 16S rRNA gene have previously been documented (Rintala et al., 2017; Lopez Leyva et al., 2021). The majority of studies used OTUs and the 2013 Greengenes database, however we recommend the use of denoising algorithms such as DADA2 or Deblur to generate ASVs or sOTUs, respectively with single nucleotide resolution and the use of updated databases. This has been discussed in detail elsewhere (Nearing et al., 2021). Strict filtering of reads post-classification and decontamination should also be performed to remove potential contaminants from further analysis (McKnight et al., 2019; Reitmeier et al., 2021).

### Ig index and other methods of analysis

Various methods were used to analyse Ig-Seq data. While some studies only reported relative abundance, others used some form of differential abundance analysis to compare differences between groups or fractions. A few Ig indices have also been proposed in analysing the differences between the composition of Ig-bound and unbound taxa. An IgA index was used in Dzidic et al. (2017) and Dzidic et al. (2020) (first used in Bunker et al., 2015), with the log of the ratio of the abundance of a taxon between the IgA+ and − fractions being measured (log (IgA+/IgA−)). This is a modification of the “Palm index” (Jackson et al., 2021) used in Palm et al. (2014), which did not use a logarithmic transformation of the ratio of relative abundance values between the IgA+ and IgA− fractions for each taxon (IgA+/IgA−). A different index was developed by Kau et al. (2015) as a measure of overall binding of a taxon and is based on relative abundance of IgA+ and IgA− fractions ( $-\frac{\log\left({IgA}^{+}+c\right)-\log\left({IgA}^{-}+c\right)}{\log\left({IgA}^{+}+c\right)+\log\left({IgA}^{-}+c\right)}$ , where *c* is a pseudo-count). Termed the “Kau index” by Jackson et al. (2021), this IgA index is an improvement of the Palm index, by using a logarithmic transformation and taking into consideration the difference of the IgA+ and − abundances relative to the total (Kau et al., 2015). This also provides directionality in the interpretation of the Ig index, with positive scores indicating greater abundance of a taxon in the Ig+ fraction and negative scores indicating greater abundance in the Ig− fraction.

Two different measures were proposed by Jackson et al. (2021) that are available in the “IgAScores” R package (https://github.com/microbialman/IgAScores), to account for the total reads in the pre-sort fraction. The IgA+ probability is the likelihood that enough of a taxon is bound by IgA such that it is able to appear in the IgA+ fraction ( ${Prob}_{ij}^{IgA+}=\frac{IgA_{ij}^{+}{FracSize}_{j}^{IgA+}}{PreSort_{ij}}$ or $\frac{IgA_{ij}^{+}{FracSize}_{j}^{IgA+}}{\max\{{PreSort}_{ij},{IgA}_{ij}^{+}{FracSize}_{j}^{IgA+}\}}$ ), where *i* is a taxon in sample *j*, *FracSize* is the fraction of IgA+ sorted bacteria, and *PreSort* is the pre-sort taxon abundance) (Jackson et al., 2021). It requires the pre-sort abundances and the percentage of IgA-bound sorted bacteria in the FACS run (Jackson et al., 2021). The advantage is that this measure can be used if the aim is only to look at bacteria with high proportions of Ig binding, and sequencing of the Ig− fraction is not required (Jackson et al., 2021). The disadvantage is that it requires FACS to be used for sorting or for flow cytometric analysis post-MACS to determine the percentage of sorted bacteria that are Ig+. It is also affected by purity of the sorted fractions, especially in the case of column-based MACS, where lower purity of sorted fractions is seen (Jackson et al., 2021). The second measure is the IgA probability ratio, which is the ratio of the IgA+ probability to the IgA− probability ( ${ProbRatio}_{ij}=\log2\left(\frac{\left({IgA}_{ij}^{+}{FracSize}_{j}^{IgA+}\right)+c}{\left({IgA}_{ij}^{-}{FracSize}_{j}^{IgA-}\right)+c}\right)/\log2\left(\frac{1+c}{c}\right)$ ), where the ratio of the IgA+ and IgA− probabilities is divided by a scaler such that *ProbRatio* values range from −1 to 1) (Jackson et al., 2021). This can distinguish taxa with lower relative Ig binding and is more sensitive than the IgA+ probability alone. An additional advantage is that sequencing of the pre-sort fraction is not required, while pre-sort differences are accounted for (Jackson et al., 2021). However, sequencing of the Ig− fraction is required to determine taxon abundance in the Ig− fraction. We will refer to these measures as the Ig+ probability and Ig probability ratio in this paper for general application to other Ig isotypes. These measures are advantageous over other previous Ig indices by accounting for pre-sort differences that may affect significance of group comparisons, and having a significantly lower coefficient of variation with more consistent results (Jackson et al., 2021). Between the two measures, the probability ratio was more robust to pre-sort differences than the Ig+ probability and had a significantly lower coefficient of variation (Jackson et al., 2021).

While papers have used relative abundance or differential abundance analysis of each taxon, the Ig probability ratio or the Ig+ probability are promising approaches for consideration in analysis of Ig-Seq data. It has been reported that the same taxon can be both Ig-bound and -unbound when using relative abundance (Ding et al., 2022). While this can also be caused by differences in Ig-binding patterns at the lower taxonomic levels (Planer et al., 2016), it was found that even the same species can be in both Ig+ and Ig− fractions (Ding et al., 2022). While acknowledging the limitations of 16S gene sequencing in resolving species-level classifications (Janda and Abbott, 2007), I–microbiota associations are mostly low-affinity and the likelihood of binding of a taxon is based on probability. The goal of Ig-Seq is therefore to look at the overall relative preference of Ig binding of taxa, and the Ig probability ratio and Ig+ probability are able to do so, while relative abundance measures are less informative. The Ig probability ratio and Ig+ probability have an advantage over differential abundance measures by taking into account pre-sort abundances and relative abundances of taxa in the same formula. We believe that the Ig probability ratio can be considered for use where possible, or the Ig+ probability as a second option in the analysis of Ig-Seq data (Figure 2). Where multiple Ig isotypes are used in a four-way sort, it is possible to use the Ig probability, followed by linear mixed effect modelling to account for the FACS quadrant (Conrey et al., 2023). Use of standardised measures as suggested will also contribute to consistency in results, which are comparable across studies, which is currently missing in the field.

### Comparing sorted fractions to the overall microbiota

In comparing the common overall unsorted microbiota to the sorted fractions across studies, we found differences in taxa after sorting. Various taxa could only be found in the overall fraction and lost after sorting while others were enriched after sorting (Table 1). Differences might be explained from processing of samples for sorting resulting in loss of bacteria from the sorted fractions. Overall, *Bifidobacterium* was found in sorted fractions in all three sample types. This suggests a possibility for enrichment of *Bifidobacterium* in the sorted fractions and a high affinity for Ig.

A possibility of a sorting bias resulting in enrichment of Alphaproteobacteria in sorted fractions was suggested by one study (Janzon et al., 2019), due to loss of bacteria that aggregate in the sorting process. However, we do not find this to be the case when comparing taxa found to be recurrent across studies according to sample type. The post-sort fractions are more likely to be enriched in certain taxa based on high affinity of binding to Ig, with higher confidence in infant stool results where multiple studies report similar findings despite differences in analysis methods. It is likely that the enrichment of Alphaproteobacteria after sorting is study specific or due to insufficient filtering of sequencing reads, and we recommend that stringent filtering and contaminant controls are employed to reduce enrichment of possible contaminants.

### Type of Ig and characterisation of Ig–microbiota associations

In comparison of binding based on Ig isotype, three studies that investigated binding of multiple isotypes were referred to. Overall, IgA bound a greater percentage of microbes than IgM and IgG (Gopalakrishna et al., 2019). While there was a large overlap of microbes bound by IgA, IgM and IgG, IgA, and IgM were more similar in binding patterns compared to IgG, which bound a subset of microbes (Janzon et al., 2019). This is in agreement with another study in mice, where both studies concluded that the difference in binding specificity to microbes in IgG was to target pathogens or pathobionts which escaped binding of IgA and were able to cross the epithelial barrier (Koch et al., 2016; Janzon et al., 2019). IgG is also not produced locally in the gut and tends to reflect systemic responses instead, such as targets of vaccination including *Haemophilus* in infancy (Janzon et al., 2019). In humans, the role of IgG binding of the microbiota is more accepted in the context of controlling specific members of the microbial population rather than aiding in colonisation of broad classes of bacteria. IgA is the most dominant antibody in mucosal immunity, and is the main antibody implicated in binding of microbiota (Chen et al., 2020). The similar binding specificities of IgA and IgM provide an allowance for functional redundancy, as seen in selective IgA deficiency, where IgM contributes to a degree of protection in place of IgA (Fadlallah et al., 2018; Catanzaro et al., 2019). The most important isotype to consider in the context of the microbiota is IgA, followed by IgM. IgG is also able to bind to the microbiota, but seems to be more important in the context of protection against pathogens or pathobionts.

Humans have two subclasses of IgA–IgA1 and IgA2 (Chen et al., 2020). One study compared the binding patterns of both subclasses in colostrum and infant stool (Sánchez-Salguero et al., 2021). IgA1 and IgA2 were found to target different bacteria (Sánchez-Salguero et al., 2021). IgA1-bound bacteria in colostrum included *Staphylococcus*, *Streptococcus*, and *Clostridium*, while IgA2-bound bacteria in colostrum were *Bifidobacterium*, *Lactobacillus*, and *Bacteroides*, and IgA2-bound bacteria in neonatal stool were *Bifidobacterium*, *Pseudomonas*, and *Paracoccus* (Sánchez-Salguero et al., 2021). There was no IgA1-associated microbiota in neonatal stool and 60% of IgA2-associated bacteria in neonatal stool potentially originated from IgA2-associated colostrum, according to a Source Tracker analysis (Sánchez-Salguero et al., 2021). The authors suggest that there is a role for IgA2-associated bacteria in maternal to infant transfer in early life, in the establishment of the infant microbiome (Sánchez-Salguero et al., 2021). IgA1 is more abundant in the small intestine and is sensitive to degradation by proteases in the large intestine, and is not found in stool samples (Brandtzaeg and Johansen, 2005). IgA2 is protease-resistant and predominates in the large intestine (Brandtzaeg and Johansen, 2005), and will be the main subclass of IgA in stool samples. The association of *Bifidobacterium* with IgA2 in both milk and neonatal stool samples may suggest a role of IgA2-associated bacteria in the development of the infant microbiome. Bifidobacteria are also important taxa in the infant microbiota due to their ability to utilise human milk oligosaccharides (HMOs) in breastmilk (Lawson et al., 2020; Stuivenberg et al., 2022), and transfer via maternal antibodies may be a mechanism to ensure infants obtain these beneficial microbes. Given that IgA1 is present in the small intestine, where the majority of Ig–microbiota associations are thought to occur (Bunker et al., 2015), there is a possibility that IgA1 from breastmilk is more important when transferred to the small intestine, where microbial antigens are known to be immunostimulatory (Mowat and Agace, 2014; Bunker et al., 2015; Million et al., 2018). When considering subclasses of IgA, both IgA1 and IgA2 can be found in colostrum, and the connection of IgA2-bound bacteria in the colostrum and infant stool may be of interest in infant gut development.

Some other considerations to take note of include IgA production in formula-fed infants and characteristics of Ig–microbiota associations. It was reported in one study that formula-fed infants had less than 1% of IgA-bound microbiota, except for some that had up to around 40% of IgA-bound bacteria (and could not be explained), while breast-fed infants had a mean of ~25% IgA-bound microbiota (Gopalakrishna et al., 2019). IgA is maternally transmitted through breastmilk in the first month of life before the infant produces its own IgA, and meconium from neonates before breastfeeding does not contain IgA (Gopalakrishna et al., 2019; Sánchez-Salguero et al., 2021). Infant samples in the study were collected between 7 and 40 days of life (Gopalakrishna et al., 2019), and most infants would have been unable to produce IgA if formula-fed. This is important to consider for designs in Ig-Seq studies as formula-fed infants would not be expected to have IgA-bound microbiota in the first month of life. More generally, Ig–microbiota binding is independent of the relative abundance of a taxon in the overall microbiota and of total Ig levels (Dzidic et al., 2017). This allows Ig-Seq to provide more depth in understanding of Ig–microbiota interactions that cannot be captured in current routine analyses.

### Comparing Ig+ and Ig− bacteria in different sample types

Differences in Ig-binding patterns were compared across sample types. In infant stool, several taxa were able to be found in both Ig+ and Ig− fractions. The taxa were *Bifidobacteriaceae, Streptococcaceae,* and *Bifidobacterium* (Table 2). Given that the taxa were found in several studies, it is likely that differences in binding exist at the lower taxonomic levels, as was found in the analysis of species or strains (Palm et al., 2014; Planer et al., 2016). It is also important to use measures that are discriminatory for overall relative binding in analysis to avoid having a taxon being both Ig+ and Ig−, as well as to look at the lower taxonomic levels for better resolution of taxa. *Enterobacteriaceae* were only found in the Ig+ fraction in infant stool, while *Bacteroides* was Ig− in maternal stool, and Ig+ bacteria in breastmilk were *Staphylococcus, Streptococcus,* and *Pseudomonas*. *Enterobacteriaceae* have been described as pro-inflammatory and Ig binding of the taxon in a healthy population is in line with reduced IgA binding of the taxon in disease states such as NEC (Gopalakrishna et al., 2019. Ig binding of *Enterobacteriaceae* is likely to control the population to prevent overgrowth and disease. On the other hand, *Bacteroides* has been reported to have lipopolysaccharide (LPS) which is anti-inflammatory or immune-inhibitory, which may explain its presence in the IgA− fraction (Pither et al., 2022; Li et al., 2023). While LPS is typically thought to be pro-inflammatory, studies have shown that the inflammatory status of LPS is dependent on the bacteria and may change in the host (Paciello et al., 2013; Vatanen et al., 2016; Li et al., 2023).

Across sample types, *Bifidobacterium* was only Ig+ in maternal stool and breastmilk, and both Ig+ and Ig− in infant stool, and it is possible that this is a result of age-related differences in Ig–microbiota binding patterns. The presence of *Bifidobacterium* in maternal samples corresponds with results from the WTR analysis, where it was reported that *B. longum* was the main bacterium transferred from the maternal gut to breastmilk (Qi et al., 2022). The strains *B. longum* subsp. *infantis* BT1, *B. longum* subsp. *infantis* JCM 11347, and *L. gasseri* HL75 were also common to both breastmilk and infant stool in one study (Qi et al., 2022). Given that *Bifidobacterium* Ig binding may contribute to transfer from maternal breastmilk to the infant gut, and that *Bifidobacterium* can also be transferred to the infant during delivery, IgA2-binding of *Bifidobacterium* may reflect transfer from mother to infant across sample types (Makino et al., 2013; Sánchez-Salguero et al., 2021).

### Changes in Ig–microbiota associations with time

In comparison of Ig–microbiota associations at common timepoints, a total of four timepoints for infant stool, one timepoint for maternal stool, and two timepoints for breastmilk were analysed (Table 3). In the stool of breast-fed infants, *Bifidobacterium, Escherichia,* and *Pseudomonas* were recurrent Ig-associated bacteria at 1 week of age, and there were no recurrent Ig-associated bacteria at 1 month of age. There were insufficient studies to compare Ig- bacteria at 1 week of age, and Ig- bacteria at 1 month of age were Clostridiales and Bifidobacteriales, based on two studies using relative abundance. At 12 months of age, IgA+ bacteria were *Ruminococcaceae* and *Bifidobacteriaceae*. *Bifidobacteriaceae* was also detected at 16 months of age. IgA− bacteria were *Bacteroidaceae, Ruminoccocaceae, Streptococcaceae,* and *Lachnospiraceae* at 12 months of age and *Lachnospiraceae* at 16 months of age. Ig–microbiota associations in maternal stool at 1 month postpartum were similar to the Ig-bound microbes in infant stool at 1 week of age (*Bifidobacterium* and *Escherichia)*, with an additional *Blautia*. Breastmilk contained *Bifidobacterium* in the Ig+ fraction at 1 week postpartum during the colostrum stage, which was also found in infant stool and maternal stool in the first month postpartum.

The similarities in Ig–microbiota associations among maternal stool, breastmilk, and infant stool in the first week or month of life indicate the importance of Ig-bound bacteria. *Bifidobacterium* is beneficial to the infant and able to gain a selective advantage in the microbiota with HMO degradation as previously discussed, and Ig binding of the taxa may aid in colonisation of the mucosa (Lawson et al., 2020; Stuivenberg et al., 2022). *Bifidobacterium* or *Bifidobacteriaceae* was also detected as Ig-bound even at 12 and 16 months of age in infant stool, and remains an important taxon in the child’s gut microbiome. In one study, *B. bifidum* was found to be consistently IgA-bound after 3 months of age, and this is also shown in this analysis (Planer et al., 2016). *Escherichia*, on the other hand, is potentially pathogenic and Ig binding of the microbe as transmitted from the mother may be to prevent it from entering systemic circulation, especially before weaning, when the infant’s immune system has not fully matured. (Zeng et al., 2016). This finding is reflected in other studies, where breastfeeding increased the Ig-binding of *E. coli*, and *Escherichia-Shigella* was found to be IgA-bound across maternal stool, infant stool, and breastmilk (Planer et al., 2016; Ding et al., 2022). IgA-binding of *Escherichia* was also found to decrease colonisation of the genus in an in vitro model of human microbiota (Sasaki et al., 2021), while N-glycan-mediated IgA binding conferred protection against *E. coli* O55 challenge in GF piglets (Raskova Kafkova et al., 2021). Additionally, adherent-invasive *E. coli* (AIEC) associated with Crohn’s disease induced production of IgA and specific binding of AIEC, while commensal *E. coli* did not induce IgA production or binding in mice (Tanaka et al., 2023).

There was also a shift in Ig-bound or -unbound bacteria in infant stool with time. While at 1 week of life, Ig-bound microbes consist of important commensals and pathogens, there is a high level of individuality in Ig–microbiota binding patterns at around 1 month of age, and no recurrent Ig-bound taxa were recorded. This is in agreement with the finding that Ig–microbiota binding patterns in unrelated infants were weakly correlated at 6 months of age, suggesting a lack of recurrent Ig–microbiota binding patterns early in life (Planer et al., 2016). There was also a genetic component affecting Ig–microbiota interactions, with Ig–microbiota binding patterns in twins being more similar than in unrelated individuals up to 21 months of age (Planer et al., 2016). There is a shift in the Ig-bound and -unbound microbes detected at 12 months onwards, such as *Ruminococcaceae* and *Lachnospiraceae*, corresponding to a shift in predominant microbes found in the infant microbiome due to transitioning from breastmilk to solid food as a dietary source (Backhed et al., 2015; McKeen et al., 2022). While there were insufficient timepoints common across studies to study Ig–microbiota binding patterns beyond 16 months of age, it was reported that Ig–microbiota binding patterns were more correlated at 2 years of age, when they resembled those of the adult (Planer et al., 2016). This again corresponds to maturation of the infant gut microbiome, and shows that the Ig–microbiota binding patterns are dynamic with time and shifts in the overall microbiome due to environmental and host factors (Backhed et al., 2015; Wernroth et al., 2022).

### Ig–microbiota binding patterns in health and disease

Differences in Ig–microbiota binding patterns were also observed between populations of health and disease. This adds greater depth to the analysis and may explain the development of clinical disease better than the overall microbiota (Gopalakrishna et al., 2019). This was investigated in four studies on undernutrition, allergic disease, and NEC (Kau et al., 2015; Dzidic et al., 2017; Gopalakrishna et al., 2019).

Undernutrition was studied in the context of kwashiorkor in humans and using mice. *Enterobacteriaceae* were enriched in the IgA+ fraction in twins discordant for kwashiorkor as compared to concordant healthy twins at diagnosis, as well as in GF mice transplanted with stool from 21-month-old kwashiorkor twins fed undernourished diets (Kau et al., 2015). *Enterobacteriaceae* Ig binding was modifiable with a peanut-based ready-to-use-therapeutic food treatment (Kau et al., 2015). Differences in Ig–microbe associations were also found in diet alone, with *Erysipelotrichaceae* being IgA-bound in mice fed undernourished diets compared to those fed standard diets (Kau et al., 2015). The IgA index and presence of enteropathogenic *E. coli* in the microbiota were also correlated to stunting and wasting scores for clinical outcomes in kwashiorkor (Kau et al., 2015). Differences in Ig binding patterns in undernutrition were also found in another study using mice, where loss of binding of beneficial bacteria such as *Lactobacillus* and *Bifidobacterium* was found in undernourished mice in stool samples, and loss of binding of *Lactobacillus* in stool and jejunal samples in undernourished mice with additional gavage of bacterial strains to induce environmental enteric dysfunction (Huus et al., 2020). It was found that loss of IgA-*Lactobacillus* associations and resultant reduced mucosal colonisation were independent of host IgA (Huus et al., 2020). *Lactobacillus* adapts to the lack of nutrients by changing surface glycans, which is also linked to mutations in carbohydrate transport and metabolism (Huus et al., 2020). This then affects the ability of *Lactobacillus* to bind to IgA via glycan-mediated interactions and reduces mucosal colonisation (Huus et al., 2020). This shows the effect of diet and the overall microbiota on Ig–microbiota associations in undernutrition, and how glycan-mediated Ig-binding in the host is affected by microbial adaptation in undernutrition.

Differences in Ig–microbiota binding patterns were also described in children who developed allergic disease and those who remained healthy at 7 years of age (Dzidic et al., 2017). Differences in the IgA–microbiota associations were already detected early in life at 1 month of age in allergy and asthma, and were present at 12 months of age in allergy, asthma, and ARC (Dzidic et al., 2017). Differences in IgA–microbiota associations were also observed for the three different types of allergic disease, with increased binding of *Lachnospiraceae* and decreased IgA binding of *Bacteroides* in allergy, reduced IgA binding of *Escherichia/Shigella* in asthma and decreased IgA binding of *Rhodobacter* and *Bacteroides* in ARC as compared to healthy controls (Dzidic et al., 2017). Microbiota predominant in *Bacteroides* has been associated with reduced immune education in early life and resultant autoimmune disease (Vatanen et al., 2016). Reduced binding of *Bacteroides* in allergy and ARC compared to healthy individuals may reflect greater immune-inhibition from *Bacteroides* LPS affecting immune education and immune-related disease development. Probiotic supplementation in the study was treated as a confounding factor in the analysis, and there was no effect of the supplementation on IgA–microbiota binding patterns or proportions of binding in allergic disease and healthy children (Dzidic et al., 2017). The same authors followed up by studying IgA–microbiota binding patterns in the breastmilk of mothers, as they showed that the probiotic affected the overall breastmilk microbiota (Dzidic et al., 2017). Specifically, the genus *Rothia* was reduced in the probiotic group (Dzidic et al., 2017). However, there was no significant difference in IgA–microbiota associations between the breastmilk of mothers of children who were diagnosed with allergic disease or not, or between probiotic and placebo groups (Dzidic et al., 2017). It was found that reduced richness of the breastmilk microbiota was correlated with allergy development instead (Dzidic et al., 2017). As the maternal cohort was predominantly allergic, and IgA–microbiota associations are distinct from the overall microbiota composition, is it possible that this affected the breastmilk IgA–microbial interactions (Dzidic et al., 2017). Additionally, they found that *Bifidobacterium* was the main taxon implicated in possible Ig–microbiota-related vertical transmission through breastmilk, as mentioned earlier. IgA binding of *Bifidobacterium,* or its presence in the overall microbiota, was not described in the study. IgA–microbiota binding patterns differ between types of allergic disease compared to healthy populations in infant stool, but further research is needed in non-allergic maternal cohorts to determine if differences in IgA-microbiota associations also apply to breastmilk and allergic disease outcomes in children.

One study investigated the IgA–microbiota associations in the development of NEC, focusing specifically on *Enterobacteriaceae* (Gopalakrishna et al., 2019). While *Enterobacteriaceae* was enriched in the overall microbiota of infants who developed NEC, reduced IgA binding of *Enterobacteriaceae* was able to explain the clinical development of NEC better than the overall microbiota alone (Gopalakrishna et al., 2019). Increasing loss of IgA-*Enterobacteriaceae* binding was also detected prior to NEC diagnosis. Further analysis in mice showed that IgA from breastmilk was required to prevent NEC development (Gopalakrishna et al., 2019). This suggests that IgA–microbiota associations have a role in the prevention of disease, and loss of healthy associations results in disease states. *Enterobacteriaceae* are inflammatory taxa related to pathogenesis, and binding of this taxon by IgA in stool samples collected in the first month of life reinforces that pathogens are likely to be bound by IgA early in infant gut development. The transfer of IgA, able to bind to pathogenic *Enterobacteriaceae* through breastmilk, highlights the importance of breastfeeding and the passive transfer of immunity to the infant while the infant’s immune system is still developing and unable to produce its own IgA (Gopalakrishna et al., 2019). Loss of IgA-bound *Enterobacteriaceae* both precedes and is indicative of NEC development according to this study, and transfer of maternal IgA able to bind to *Enterobacteriaceae* through breastfeeding is possibly protective of infant NEC development. However, more studies are required to confirm this.

Current knowledge gaps include Ig–microbiota associations in maternal stool both pre- and postpartum, and the differences between the Ig-bound and -unbound taxa in breastmilk at different stages. Even in infant stool, there is a lack of common timepoints between 1 week and 1 month and between 1 month and 12 months. The changes in Ig–microbiota binding patterns according to critical periods of development have also not been reported. It also needs to be confirmed if the IgA2-association of microbes is the mechanism of transfer of commensals from mother to infant. Given studies in undernutrition reporting changes in surface glycans in microbes in response to nutritional pressures affecting IgA binding and mucosal colonisation, the importance of diet in Ig–microbiota associations and the ability to correct for associations using diet should also be studied. It is not known how diet changes in a healthy cohort are able to influence Ig–microbiota binding patterns, or if maternal diet is able to do so in the infant when breastfeeding. In allergic disease, the possibility of *Bifidobacterium* Ig binding in breastmilk and infant stool should be studied in a non-allergic maternal cohort. There is also a lack of understanding of how maternal and infant stool Ig–microbiota associations are related, and whether maternal Ig–microbiota associations would affect those of the infant. Future studies involving all three sample types are required to arrive at more robust conclusions. Dietary and probiotic interventions have been used in some studies included; however, while one study showed that dietary intervention was able to influence Ig–microbiota binding patterns, other studies have not shown any effect of probiotic interventions on Ig–microbiota binding patterns. More studies are required to confirm this and determine factors able to influence Ig–microbiota binding patterns to correct them in the prevention of disease development.

## Conclusion

This scoping review synthesises current evidence on Ig–microbiota binding patterns in maternal and infant contexts, highlighting consistent associations despite substantial methodological heterogeneity and a limited, early-stage evidence base. It shows that IgA is the dominant antibody in Ig-Seq studies, with partial functional overlap with IgM and a more selective role for IgG, and that Ig–microbiota binding patterns vary by sample type, Ig subclass, age, diet, and health status, evolving alongside microbiome maturation. This review suggests that sorting does not result in enrichment of Alphaproteobacteria, and differences between Ig-bound and -unbound taxa reflect relative binding affinity, with key commensals and potential pathogens showing distinct patterns across mother–infant dyads and over time. The review emphasises that Ig-Seq can reveal disease-relevant signals, sometimes preceding clinical manifestation, beyond those seen in overall microbiota profiles, but stresses that progress is constrained by small study numbers, inconsistent methods, and gaps in longitudinal, maternal–infant, dietary, and intervention data. To address these issues, we recommend standardisation of Ig-Seq terminology, laboratory methods, bioinformatic pipelines, and statistical approaches to improve reproducibility, comparability, and the translational potential of Ig–microbiota research.

## Supporting information

10.1017/gmb.2026.10028.sm001 Yi Jia et al. supplementary material Yi Jia et al. supplementary material

## Data Availability

The authors confirm that the data for this study is available within the article and in the supplementary materials.

## Supplementary material

The supplementary material for this article can be found at http://doi.org/10.1017/gmb.2026.10028.

## Long descriptions

### Figure 1. Long description

The flowchart is organized into three vertical phases labeled on the left: Identification, Screening, and Included.

Phase 1: Identification

* Left Pathway (Databases and Registers): Records identified from Databases n equals 931. Breakdown includes S C O P U S n equals 370, M E D L I N E n equals 94, Embase n equals 220, and Web of Science n equals 247. An arrow points right to Records removed before screening: Duplicate records removed n equals 318.

* Right Pathway (Other Methods): Records identified from Citation searching n equals 12.

Phase 2: Screening

* Left Pathway: Records screened n equals 614. An arrow points right to Records excluded n equals 589. Below screening, Reports sought for retrieval n equals 25. An arrow points right to Reports not retrieved n equals 0. Below retrieval, Reports assessed for eligibility n equals 25. An arrow points right to Reports excluded n equals 18, with reasons including Conference abstracts n equals 8, Previews n equals 2, Wrong age n equals 1, Culture and no sorting n equals 1, No sorting or sequencing n equals 1, No sequencing of sorted fractions n equals 3, and Mice only n equals 2.

* Right Pathway: Reports sought for retrieval n equals 2. An arrow points right to Reports not retrieved n equals 0. Below retrieval, Reports assessed for eligibility n equals 2. An arrow points right to Reports excluded n equals 0.

Phase 3: Included

* At the bottom left, a final box labeled Studies included in review n equals 9 receives arrows from both the left and right eligibility assessment boxes.

Navigate back to Figure 1..

### Figure 2. Long description

The flowchart begins at the top with a stool sample containing I g-bound microbes.

Step A: Overall microbiome sequencing of the initial sample.

Step 1: Pre-processing involving centrifugation and filtering to isolate a pre-sort fraction.

Step B: Optional sequencing of the pre-sort fraction.

Step 2: Staining with secondary antibody with fluorescent conjugate and fluorescent nucleic acid stain.

The workflow then splits into two parallel paths:

Left Path (3a to 6a): Fluorescence-activated cell sorting for purity. It involves sorting into I g plus, I g minus, and negative control fractions. This is followed by sequencing (4a), bioinformatic processing (5a), and statistical analysis (6a) to determine I g index per taxon using I g probability ratio or I g plus probability.

Right Path (3b to 6b): Magnetic-activated cell sorting for enrichment using column-based or plate-based methods. It involves sorting into I g plus, I g minus, and negative control fractions. This is followed by sequencing (4a), bioinformatic processing (5b), and statistical analysis (6b) focusing on I g plus probability and other I g-indices.

Step 7: The two paths converge for a comparison across groups, illustrated by a green silhouette of a healthy aging sequence (infant to adult) and a red silhouette of a diseased aging sequence (infant to patient in a hospital bed).

Navigate back to Figure 2..

### Table 1. Long description

The table is organized into three main sample types: Infant stool, Maternal stool, and Breastmilk. Each sample type is analyzed for Recurrent unsorted bacteria (overall microbiota) and Recurrent sorted (I g plus and I g minus combined) bacteria, categorized by Order, Family, Genus, and Study.

* Infant stool:

- Unsorted bacteria include f_Lachnospiraceae, f_Enterobacteriaceae, g_Bacteroides, g_Lactobacillus, g_Veillonella, and g_Bifidobacterium.

- Sorted bacteria include o_Bifidobacteriales, g_Bifidobacterium, g_Paracoccus, and g_Pseudomonas.

- Studies cited: Gopalakrishna et al. 2019, Janzon et al. 2019, Sánchez-Salguero et al. 2021, Ding et al. 2022, and Qi et al. 2022.

* Maternal stool:

- Unsorted bacteria include g_Blautia and g_Faecalibacterium.

- Sorted bacteria include g_Blautia, g_Bifidobacterium, g_Faecalibacterium, and g_Escherichia.

- Studies cited: Ding et al. 2022 and Qi et al. 2022.

* Breastmilk:

- Unsorted bacteria include g_Bifidobacterium, g_Pseudomonas, g_Streptococcus, and g_Staphylococcus.

- Sorted bacteria include g_Bifidobacterium.

- Studies cited: Sánchez-Salguero et al. 2021, Ding et al. 2022, and Qi et al. 2022.

Note: Italics in the original table indicate results based on relative abundance without differential analysis.

Navigate back to Table 1..

### Table 2. Long description

The table is organized into nine columns. The first column identifies the Sample type. The next four columns describe Recurrent I g plus bacteria (Order, Family, Genus, Study). The final four columns describe Recurrent I g minus bacteria (Order, Family, Genus, Study).

* Infant stool:

- Recurrent I g plus includes Family f underscore Bifidobacteriaceae (Genus g underscore Bifidobacterium), Family f underscore Streptococcaceae, and Family f underscore Enterobacteriaceae.

- Recurrent I g minus includes Family f underscore Bifidobacteriaceae (Genus g underscore Bifidobacterium), Family f underscore Streptococcaceae, and Family f underscore Lachnospiraceae.

* Maternal stool:

- Recurrent I g plus includes Genus g underscore Escherichia, g underscore Bifidobacterium, and g underscore Blautia.

- Recurrent I g minus includes Genus g underscore Bacteroides.

* Breastmilk:

- Recurrent I g plus includes Genus g underscore Staphylococcus, g underscore Streptococcus, g underscore Bifidobacterium, and g underscore Pseudomonas.

- Recurrent I g minus is marked as Insufficient studies.

Studies cited include Planer et al. 2016, Janzon et al. 2019, Sanchez-Salguero et al. 2021, Ding et al. 2022, Dzidic et al. 2017, Kau et al. 2015, and Qi et al. 2022.

Navigate back to Table 2..

### Table 3. Long description

The table is divided into three main sample types: Infant stool, Maternal stool, and Breastmilk.

1. Infant stool:

* 1 week of age: Recurrent I g plus bacteria include Genus Bifidobacterium, Escherichia, and Pseudomonas. I g minus data is noted as insufficient studies.

* 1 month of age: No recurring taxa for I g plus. I g minus bacteria include Order Clostridiales and Bifidobacteriales.

* 12 months of age: Recurrent I g plus include Family Ruminococcaceae and Bifidobacteriaceae. Recurrent I g minus include Family Bacteroidaceae, Ruminococcaceae, Streptococcaceae, and Lachnospiraceae.

* 16 months of age: Recurrent I g plus include Family Bifidobacteriaceae. Recurrent I g minus include Family Lachnospiraceae.

2. Maternal stool at 1 month postpartum:

* Recurrent I g plus bacteria include Genus Bifidobacterium, Blautia, and Escherichia. I g minus data is noted as insufficient studies.

3. Breastmilk:

* 1 week postpartum (colostrum stage): Recurrent I g plus include Genus Bifidobacterium. I g minus data is noted as insufficient studies.

* 1 month postpartum (mature milk stage): No recurring taxa for I g plus. I g minus data is noted as insufficient studies.

Taxonomic levels are prefixed with o underscore for Order, f underscore for Family, and g underscore for Genus.

Navigate back to Table 3..
